# A Novel Change Detection Method Based on Statistical Distribution Characteristics Using Multi-Temporal PolSAR Data

**DOI:** 10.3390/s20051508

**Published:** 2020-03-09

**Authors:** Jinqi Zhao, Yonglei Chang, Jie Yang, Yufen Niu, Zhong Lu, Pingxiang Li

**Affiliations:** 1 State Key Laboratory of Information Engineering in Surveying, Mapping and Remote Sensing, Wuhan University, Wuhan 430079, China; masurq@whu.edu.cn (J.Z.); yangj@whu.edu.cn (J.Y.); pxli@whu.edu.cn (P.L.); 2 Jiangsu Key Laboratory of Resources and Environmental Information Engineering, China University of Mining and Technology, Xuzhou 221116, China; 3 Faculty of Geomatics, East China University of Technology, Nanchang 330013, China; 4 College of Geology Engineering and Geomatics, Chang’an University, Xian 710054, China; 2015026021@chd.edu.cn; 5 Huffington Department of Earth Sciences, Southern Methodist University, Dallas, TX 75275, USA; zhonglu@smu.edu

**Keywords:** change detection, omnibus test statistic, Kittler and Illingworth (K&I), Weibull distribution, gamma distribution, PolSAR

## Abstract

Unsupervised change detection approaches, which are relatively straightforward and easy to implement and interpret, and which require no human intervention, are widely used in change detection. Polarimetric synthetic aperture radar (PolSAR), which has an all-weather response capability with increased polarimetric information, is a key tool for change detection. However, for PolSAR data, inadequate evaluation of the difference image (DI) map makes the threshold-based algorithms incompatible with the true distribution model, which causes the change detection results to be ineffective and inaccurate. In this paper, to solve these problems, we focus on the generation of the DI map and the selection of the optimal threshold. An omnibus test statistic is used to generate the DI map from multi-temporal PolSAR images, and an improved Kittler and Illingworth algorithm based on either Weibull or gamma distribution is used to obtain the optimal threshold for generating the change detection map. Multi-temporal PolSAR data obtained by the Radarsat-2 sensor over Wuhan in China are used to verify the efficiency of the proposed method. The experimental results using our approach obtained the best performance in East Lake and Yanxi Lake regions with false alarm rates of 1.59% and 1.80%, total errors of 2.73% and 4.33%, overall accuracy of 97.27% and 95.67%, and Kappa coefficients of 0.6486 and 0.6275, respectively. Our results demonstrated that the proposed method is more suitable than the other compared methods for multi-temporal PolSAR data, and it can obtain both effective and accurate results.

## 1. Introduction

Change detection is an important remote sensing technology that is used to identify the changes of the Earth’s surface through multi-temporal images of the same geographical area observed at different times [1]. On the one hand, due to the impact of environmental factors and social development, changes occur all the time. On the other hand, as a result of the development of satellite systems, a huge number of remote sensing images can now be acquired to detect these changes. Owing to the explosive increase in remote sensing data, how to detect changes accurately is an active research topic [2]. In this context, multi-temporal remote sensing images of the same region proved particularly useful in the applications of change detection, including urban planning [3,4], agricultural research [5,6], disaster detection [7,8], and wetland detection [9,10]. As one of the effective means in change detection, synthetic aperture radar (SAR) systems have more advantages than optical sensors, due to their ability to acquire periodic images, regardless of weather or daylight. 

Unsupervised change detection methods [11,12] have the advantage of a simple design, and they involve the following steps: (1) data preprocessing, (2) production of the difference image (DI), and (3) analysis of the DI. The main techniques used in the first step are radiometric calibration, image co-registration [13], and speckle filtering [14]. Precise preprocessing is vital to improve the accuracy of the change detection results. After the preprocessing, the quality of the DI map and the choice of threshold algorithm are closely linked to the results of the unsupervised change detection. In the second step, many different approaches, both pixel-based and object-based, can be used to produce a DI map, and they each have their own respective advantages and disadvantages [15]. The pixel-based approaches are easy to design and quick to process. As such, they are widely used in generating DI maps [16]. In contrast, the object-based approaches combine the pixels of homogeneous regions, which can preserve the detailed information [17,18]. However, these methods require many parameters to be set. Parameter testing is a key factor that influences the performance of DI maps, and it results in the object-based approaches being inefficient. In order to design a suitable and efficient approach, pixel-based methods are the focus in our research. To date, many different approaches were developed for producing DI maps, such as the log-ratio technique [19,20], the neighborhood-based ratio technique [21], feature fusion [22,23], Markov random field-based models [24,25], principal component analysis [26], Kullback–Leibler divergence method [27], and time-series analysis on Google Earth Engine [28,29]. However, these methods only use single/dual polarization SAR data. In consideration of the special distribution of SAR backscattering [30] and using more channel information, the test statistic method with maximum likelihood estimation (MLE) [31], change detection matrices with Wishart distance [32], Kennaugh element framework [33], and the Hotelling–Lawley trace statistic [11] can apply the covariance (or coherence) matrix to obtain DI maps and fit for detecting the abrupt changes. As an extension of the test statistic, the omnibus likelihood ratio test statistic approach, which can be applied in dual-/quad-pol acquisitions [34,35], proved particularly useful in multi-temporal change detection, and it can detect both abrupt changes and steady increased changes [35]. In this paper, multi-temporal covariance (or coherence) matrices are still satisfied with a Wishart distribution and used by the omnibus likelihood ratio test statistic approach to produce an accurate DI map. Finally, in order to choose the threshold accurately and efficiently, a number of methods were developed based on automatic threshold selection [31,36,37]. Compared with these methods, the Kittler and Illingworth (K&I) algorithm is based on Bayesian theory, and it determines the optimal threshold by minimizing the classification error. This approach has a solid mathematical foundation and is widely used in image processing. However, one problem that should be noted is that the pre-assumed distribution model for the DI has a great impact on the effectiveness of the K&I algorithm. In the previous studies of change detection, the DIs were usually generated from single-channel SAR images, and Gaussian, generalized Gaussian, Rayleigh, or exponential distribution models were assumed for the statistical property of the DI [38,39,40]. However, as soon as a DI map is generated by the omnibus likelihood ratio test statistic method with four channels of polarimetric synthetic aperture radar (PolSAR) data, the statistical property of the DI is changed. In order to obtain accurate change detection results, a more appropriate distribution model should be adopted and, consequently, new versions of the K&I algorithm need to be developed. Weibull and gamma distributions are widely used in analyzing the statistical characterization of SAR data [41], and they are applied in image segmentation [42], texture analysis [43], deformation modeling [44], classification [45], and target detection [46]. However, to our knowledge, Weibull or gamma distribution was not previously applied to describe the statistical behaviors of a change detection DI map. Therefore, in order to obtain a precise threshold, Weibull or gamma distribution is used to modify the K&I algorithm.

Above all, we propose a novel change detection method based on the omnibus likelihood ratio test statistic and improved K&I algorithm using the Weibull or gamma distribution in this paper. The appropriate DI map is generated by an omnibus likelihood ratio test statistic from the multi-temporal covariance (or coherency) matrix. Moreover, to estimate the distribution properties of the DI map, statistical histogram curve-fitting is utilized to determine the distribution model. Furthermore, Weibull or gamma distribution is used to improve the K&I algorithm, as well as obtain the optimal threshold. Finally, an accurate change detection map is generated using the DI map and the optimal threshold.

The rest of this paper is organized as follows: the principle and detailed procedures of the proposed method are described in Section 2. Section 3 introduces the case study. Section 4 provides the experimental results and analyses. Potentials and limitations of the proposed method are briefly discussed in Section 5. Finally, our conclusions are drawn in Section 6.

## 2. Materials and Methods

### 2.1. The Model of PolSAR Data

Fully polarimetric information is used in the proposed method, which can be expressed by Equation (1) when the target reciprocity condition is satisfied.

(1) $$\Omega ={\left[S_{hh},\sqrt{2}S_{hv},S_{vv}\right]}^{T},\;$$

where *h *and *v *represent horizontal and vertical polarization, respectively. The scattering vector *S_hv_*represents the vertical transmitting and horizontal receiving polarizations. *T *denotes the transpose operation. 

After multi-look processing, as an example of a covariance matrix, the backscattered information matrix of the ground target follows a Wishart distribution and can be expressed as follows:

(2) $$X=\langle \Omega \cdot \Omega^{*T}\rangle =\langle \left[\begin{array}{ccc} {\left|S_{hh}\right|}^{2} & S_{hh}S_{hv}^{*} & S_{hh}S_{vv}^{*}\\ S_{hv}S_{hh}^{*} & {\left|S_{hv}\right|}^{2} & S_{hv}S_{vv}^{*}\\ S_{vv}S_{hh}^{*} & S_{vv}S_{hv}^{*} & {\left|S_{vv}\right|}^{2} \end{array}\right]\rangle .$$

After image preprocessing, the co-registered and equal-sized PolSAR images from the same geographical region can be represented by *X_t1_* (*i, j*), *X_t2_* (*i, j*), …, *X_tn_* (*i, j*) at time *t1*, *t2, *…, *tn*, respectively. Because of the different data obtained over a long time interval, we can assume that the temporal PolSAR measurements (*X_t1_*, *X_t2 _*…*X_tn_*) are independent and can be expressed as follows:

(3) $$\begin{array}{c} X_{t1}\in W(p,m_{1},\Sigma_{X_{t1}})\\ X_{t2}\in W(p,m_{2},\Sigma_{X_{t2}})\\ \vdots \\ X_{tn}\in W(p,m_{n},\Sigma_{X_{tn}}) \end{array},$$

where *p* represents the matrix dimension of* X_t1_*, *X_t2_, *…, *X_tn_*, and *m_1_*, *m_2_, *…, *m_n_* represents the number of looks of *X_t1_*, *X_t2_, *…, *X_tn_*, respectively. The probability density function (PDF) of the *X_ti_* is expressed by Equation (4). *Σ_Xti_* denotes the dispersion matrix of the *i-*th PolSAR image and is calculated by Equation (5).

(4) $$f(X_{ti})=\frac{1}{\upsilon_{p}\left(m_{i}\right)}{\left|\Sigma_{X_{ti}}\right|}^{-m_{i}}{\left|X_{ti}\right|}^{m_{i}-p}\exp\left\{-tr\left[\Sigma^{-1}X_{ti}\right]\right\},$$

where $\upsilon_{p}\left(m_{i}\right)=\pi^{p(p-1)/2}\prod_{j=1}^{p}\Gamma (m_{i}-j+1)$.

(5) $$\Sigma_{X_{ti}}=\frac{1}{m_{i}}X_{ti}.$$

### 2.2. The Proposed Method

#### 2.2.1. Omnibus Test Statistic 

When a change occurs in the time interval [*t1, tn*], we need to check these changes by comparing the PolSAR measurements. The omnibus test statistic [35], which has proved useful in detecting both discontinuous jump and steady increased changes, is introduced in this section.

Comparing *X_t1_*, *X_t2_*, …, *X_tn_*, when these dispersion matrices satisfy *Σ_Xt1_ = Σ_Xt2_ = *… *= Σ_Xtn_*, this means that the ground targets in these regions at different dates show the characteristic of being unchanged, and they can be defined by the null hypothesis (*H_0_*). In contrast, when these dispersion matrices satisfy *Σ_Xti _*≠ *Σ_Xtj_* and *i*, *j* denote the arbitrary time, this means that at least one change happened in these regions, and it can be defined by the alternative hypothesis (*H_1_*). 

The joint densities of omnibus test statistics based on MLE can be expressed by *f *(*Σ_Xt1_, Σ_Xt2_, *…, *Σ_Xti_, ε*), where the parameter *ε* is the set of the probability function. Furthermore, the omnibus test statistic (*Q*) using the likelihood ratio is expressed by Equation (6).

(6) $$Q=\frac{\max_{\theta \in H_{0}}L(\varepsilon )}{\max_{\theta \in \Omega }L(\varepsilon )}=\frac{L\left(\hat{\Sigma }\right)}{\prod_{i=1}^{n}L_{X_{ti}}\left(\Sigma_{X_{ti}}\right)},\;$$

where *L*(▪) and *f*(▪) are the likelihood function and frequency function, respectively, and Ω = *H_0_***∪***H_1_*. When combined with Equations (3) and (5), $\hat{\Sigma }$ can be expressed as follows:

(7) $$\hat{\Sigma }=\frac{1}{\sum_{i=1}^{n}m_{i}}\sum_{i=1}^{n}X_{ti}.$$

If we assume that *Σ_Xt1 _*= *Σ_Xt2_* = … =*Σ_Xtn_*=*Σ*, and Equation (4) is input into Equation (6), then *Q* can be expressed as follows:

(8) $$Q=\frac{\frac{{\left|\hat{\Sigma }\right|}^{-\sum_{i=1}^{n}m_{i}}}{\prod_{i=1}^{n}\Gamma_{p}\left(m_{i}\right)}\prod_{i=1}^{n}{\left|X_{ti}\right|}^{m_{i}-p}\exp\left\{-tr\left[\Sigma^{-1}\left(\sum_{i=1}^{n}X_{ti}\right)\right]\right\}}{\prod_{i=1}^{n}\left(\frac{1}{\Gamma_{p}\left(m_{i}\right)}{\left|\Sigma_{X_{ti}}\right|}^{-m_{i}}{\left|X_{ti}\right|}^{m_{i}-p}\exp\left\{-tr\left[\Sigma^{-1}X_{ti}\right]\right\}\right)}.$$

When combined with Equation (7) and assuming *m_1_* = *m_2_*= … = *m_n_*= *m*, Equation (8) can be simplified as follows:

(9) $$Q={\left\{n^{pn}\frac{\prod_{i=1}^{n}\left|X_{ti}\right|}{{\left|X_{ti}\right|}^{n}}\right\}}^{m}.$$

After a logarithm operation, the DI map (*d*) with the omnibus test statistic can be denoted by Equation (10).

(10) $$d=\frac{2p^{2}-4pm-1}{2p}(pn\ln n+\sum_{i=1}^{n}\ln\left|X_{ti}\right|-n\ln\left|\sum_{i=1}^{n}X_{ti}\right|).$$

#### 2.2.2. The K&I Algorithm Based on Weibull or Gamma Distribution

(a) Statistical Distribution Model of the DI Map

After generating the DI map from the multi-temporal PolSAR images, an automatic thresholding method is introduced. To obtain good change detection results, an appropriate thresholding algorithm is needed to separate the DI into the changed and unchanged classes. We recommend the K&I algorithm, which obtains the optimal threshold value by minimizing the classification error [38]. As the statistical distribution function is considered in this algorithm, it is best to establish which distribution model is most compatible with the DI map. Typically, Gaussian and generalized Gaussian distribution models are used in remote sensing imagery. Due to the impact of speckle noise, the negative exponential model is widely used in SAR imagery [47]. Furthermore, the Weibull distribution can be compatible with PolSAR data over a wide resolution range, and the PDF involves a scale parameter *θ* and a shape parameter *γ*, as follows:

(11) $$p\left(X\right)=\frac{\gamma }{\theta }X^{\gamma -1}\exp\left(-\frac{X^{\gamma }}{\theta }\right),X>0.$$

The gamma distribution is also a two-parameter distribution model, which can degenerate to an exponential or chi-squared distribution in some circumstances. Moreover, this distribution model is fit for PolSAR imagery with a medium resolution. The PDF of the gamma distribution involves a rate parameter *θ*, a shape parameter *γ*, and a gamma function Г(*γ*), as follows:

(12) $$p\left(X\right)=\frac{\theta^{\gamma }X^{\gamma -1}e^{-\theta X}}{\Gamma \left(\gamma \right)},X\geq 0.\;$$

A visual way to determine if the distribution model is suitable or not is distribution function fitting. After we make the distribution function fit in some homogeneous areas via MLE operation, we can observe the goodness of fit by comparing it with the statistical histogram. The homogeneous areas can be lakes or vegetation areas. Moreover, as one of important types of surface coverage, urban area was also selected to analyze its distribution property.

(b) The K&I Algorithm Based on Weibull or Gamma Distribution

In order to obtain a precise threshold, both Weibull and gamma distributions are separately used to modify the K&I algorithm. As an extension of Bayesian theory, the traditional K&I thresholding method can be expressed as shown in Equation (13),

(13) $$J(T)=\sum_{d_{l}=0}^{L-1}h(d_{l})c(d_{l},T)\;\mathrm{where}\;c(d_{l},T)=\left\{\begin{array}{l} -2\ln P\left(\omega_{u}|d_{l},T\right),d_{l}\leq T\\ -2\ln P\left(\omega_{c}|d_{l},T\right),d_{l}>T \end{array}\right.$$

where *L*, *T, *and* h(d_l_) *denote the numbers of possible gray levels, the threshold, and the histogram of the DI map, respectively. *J*(▪) and* c*(▪) denote the criterion function and cost function, respectively. Under the condition of* d_l_* and the threshold* T, P*(*ω_i_|d_l_,T*) (*i *= *u,c*) denotes the posterior probability of unchanged (*u*) and changed (*c*) classes, respectively.

The traditional K&I method assumes that the class-conditional distribution follows a Gaussian, generalized Gaussian, Rayleigh, or exponential distribution, but this assumption cannot accurately reflect the distribution of the DI map. After we analyzed the statistical property of the DI, we found that it was more compatible with the two-parameter distribution models, i.e., Weibull or gamma distributions, which is later confirmed in the experimental part. In order to improve the threshold selection, an improved model based on Weibull or gamma distribution is used to describe the statistical behaviors of the changed and unchanged classes in the DI. We, therefore, propose a new and improved version of the K&I algorithm. If we assume that the distribution model is a Weibull distribution, by combining Equations (11) and (13), the new criterion function can be shown as follows:

(14) $$\begin{array}{ccc} J\left(T\right) & = & \sum_{d_{l}=0}^{T}h\left(d_{l}\right)\left\{-\ln P_{u}-\ln\left(\frac{\gamma_{u}}{\theta_{u}}d_{l}{}^{\gamma_{u}-1}\exp\left(-\frac{d_{l}{}^{\gamma_{u}}}{\theta_{u}}\right)\right)\right\}\\ & + & \sum_{d_{l}=T+1}^{L-1}h\left(d_{l}\right)\left\{-\ln P_{c}-\ln\left(\frac{\gamma_{c}}{\theta_{c}}d_{l}{}^{\gamma_{c}-1}\exp\left(-\frac{d_{l}{}^{\gamma_{c}}}{\theta_{c}}\right)\right)\right\} \end{array}$$

The symbols *θ_u_*, *γ_u_*, *θ_c_* and *γ_c_* are the scale and shape parameters of *P_u_*(*d_l_*| *u, T*) and *P_c_*(*d_l_*| *c, T*), respectively, which are the Weibull distribution functions estimated with the pixels in the DI map by MLE. After expanding the above equation, the criterion is changed to

(15) $$\begin{array}{cc} J\left(T\right)= & \sum_{d_{l}=0}^{T}h\left(d_{l}\right)\left(d_{l}{}^{\gamma_{u}}/\theta_{u}\right)-\sum_{d_{l}=0}^{T}h\left(d_{l}\right)\ln\left(d_{l}{}^{\gamma_{u}-1}\right)-P_{u}\ln\left(P_{u}\cdot \gamma_{u}/\theta_{u}\right)+\\ & \sum_{d_{l}=T+1}^{L-1}h\left(d_{l}\right)\left(d_{l}{}^{\gamma_{c}}/\theta_{c}\right)-\sum_{d_{l}=T+1}^{L-1}h\left(d_{l}\right)\ln\left(d^{\gamma_{c}-1}\right)-P_{c}\ln\left(P_{c}\cdot \gamma_{c}/\theta_{c}\right) \end{array}$$

When the gamma distribution is considered, we can generate another criterion function by combining Equations (12) and (13). Following the same operation as above, the K&I algorithm based on a gamma distribution is as follows:

(16) $$\begin{array}{ccc} J\left(T\right) & = & \sum_{d_{l}=0}^{T}h\left(d_{l}\right)\left(\theta_{u}d_{l}-\ln\left(d_{l}{}^{\gamma_{u}-1}\right)\right)-P_{u}\ln\left(P_{u}\cdot \theta_{u}{}^{\gamma_{u}}/\Gamma \left(\gamma_{u}\right)\right)\\ & + & \sum_{d_{l}=T+1}^{L-1}h\left(d_{l}\right)\left(\theta_{c}d_{l}-\ln\left(d_{l}{}^{\gamma_{c}-1}\right)\right)-P_{c}\ln\left(P_{c}\cdot \theta_{c}{}^{\gamma_{c}}/\Gamma \left(\gamma_{c}\right)\right) \end{array}$$

In the application of this algorithm, we firstly calculate the statistical histogram of the DI. We then compute criterion *J*(*T*) at each histogram level. Finally, we choose the histogram level where criterion *J*(*T*) is the minimum as the optimal threshold, i.e., T^*^= ***argmin***{*J*(*T*): *T *= 0, 1, …, *L *− 1}. In change detection, the area is marked as a changed one if its pixel value is higher than the optimal threshold in the DI.

#### 2.2.3. The Workflow of the Proposed Method

**Figure 1:**
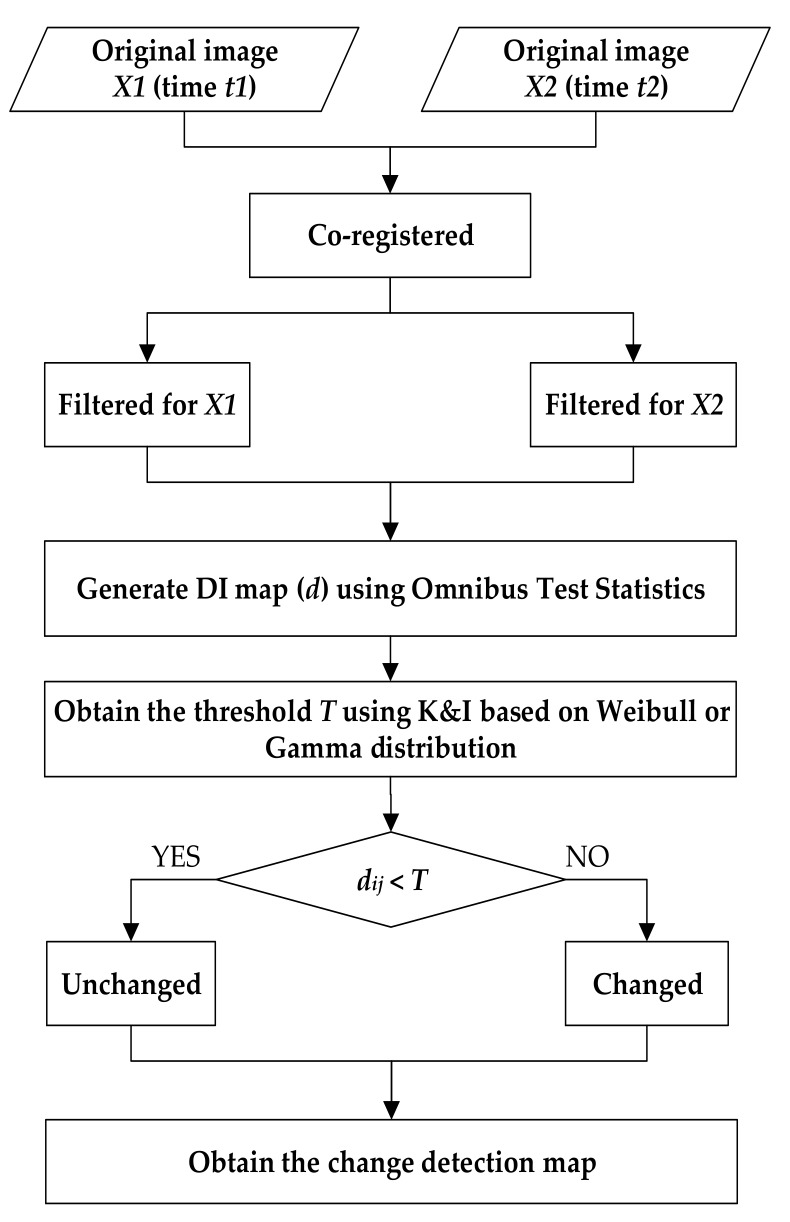
Flowchart of the proposed method.

The entire steps in the proposed approach can be demonstrated as follows:

- Step (1): The preprocessing that involves co-registration and removal of speckle noise for the test data is firstly conducted. In this study, the precision of the co-registration was less than one pixel, and the refined Lee filter [47] was used to suppress the speckle noise.
- Step (2): The omnibus test statistic method is used to generate the DI map from PolSAR images at different dates.
- Step (3): Statistical histogram curve-fitting is utilized to determine the function distribution model in homogeneous and unchanged regions. Weibull and gamma distributions are separately applied to improve the K&I algorithm.
- Step (4): The pixels in position (*i, j*) at different dates should be determined as unchanged or changed. If *d_ij_* < *T*, the index of this pixel is equal to zero, which denotes that the pixels in this position are similar or unchanged. Otherwise, the corresponding pixels are deemed as different or changed, and the index is equal to one. After completing the indexes of all the pixels in the multi-temporal images, the change detection map is produced. Figure 1 shows the detailed algorithm flowchart.

### 2.3. Evaluation Criterion

For the quantitative evaluation, the false alarm (FA) rate, the total errors (TE), the overall accuracy (OA), and the Kappa coefficient are used to verify the performance of the proposed method. When the ground truth is available, these indicators can be expressed as follows:

(17) $$\left\{\begin{array}{l} FA=\frac{FP}{N_{u}},TE=\frac{FP+FN}{N}\\ OA=\frac{TP+TN}{N},Kappa=\frac{OA-Pe}{1-Pe}\\ Pe=\frac{\left(TP+FN)(TP+FP)+(FP+TN)(FN+TN\right)}{N^{2}}\\ N=N_{c}+N_{u} \end{array}\right.,$$

where true positives (TP) denote the number of changed points which are detected correctly, true negative (TN) denotes the number of unchanged points which are detected correctly, false positives (FP) denote the number of unchanged points which are detected incorrectly, and false negatives (FN) denote the number of changed points which are detected incorrectly. Moreover, the numbers of changed points and unchanged points are denoted by* N_c_* and* N_u_*, respectively.

## 3. Case Study

### 3.1. Study Area and Background

**Figure 2:**
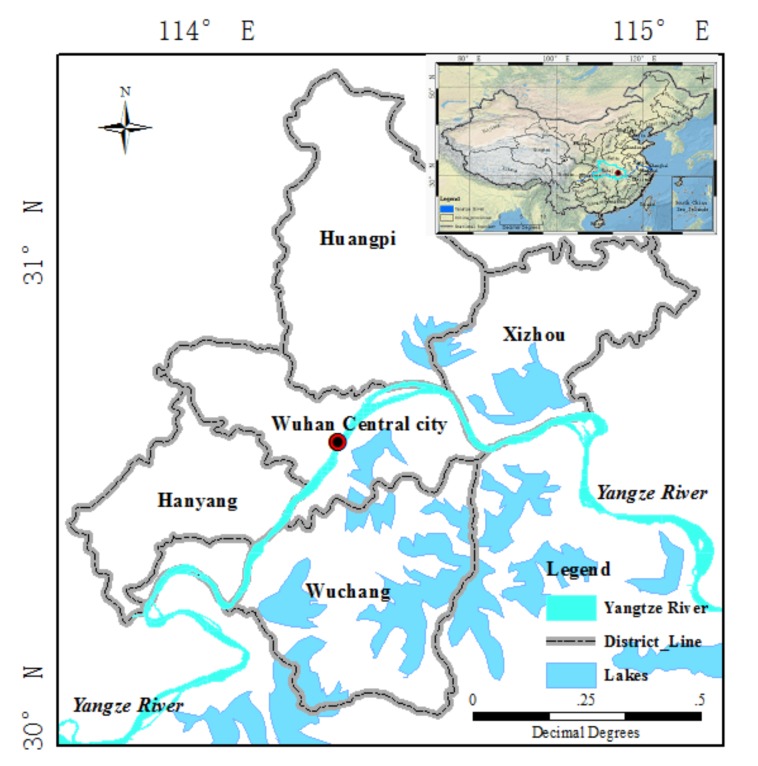
Location of the research area.

As a central city in China, the city of Wuhan is located in the eastern part of Hubei province and lies in the middle reaches of the Yangtze River (Figure 2). It belongs to the north subtropical monsoon climate zone, and it has abundant rainfall. There are many rivers and lakes in Wuhan, which account for one-quarter of the total city area. Due to urbanization and population growth, Wuhan is one of the most rapidly developing cities in China. As a result, how to detect changes accurately is important for both regional economic development planning and dynamic monitoring. Because of the construction of the East Lake Greenway and the 50-year return period rainfall that occurred between 2015 and 2016, changes in urban facilities and inundation detection are the emphasis of this research. Compared with optical sensors, SAR sensors are a better solution to detect these changes in the scenario of constant rainfall. Two PolSAR images of Wuhan from this period were, thus, used to monitor the changes in urban facilities and those caused by inundation.

### 3.2. Datasets and Image Preprocessing

Because this flooding event happened suddenly and receded rapidly, the timing of SAR data acquisition is extremely critical. Thanks to the C-band Radarsat-2 sensor (with a repeat cycle of 24 days), we acquired near-real-time PolSAR data on 6 July 2016. Furthermore, the archived pre-event PolSAR data were used to detect changes caused by urbanization and inundation in Wuhan. Table 1 shows the parameters of the experimental datasets. 

In order to ensure the precision of the change detection results, the preprocessing of the experimental datasets included radiometric calibration, image co-registration, and speckle filtering, all of which were completed using two free open-source software packages (NEST and PolSARpro). After the preprocessing, the pixel values at the different dates were directly related to the radar backscattering coefficients and had the same original resolution. Moreover, for the co-registered images, speckle noise was removed by refined Lee filtering based on a 7 × 7 window.

## 4. Results and Analyses

**Figure 3:**
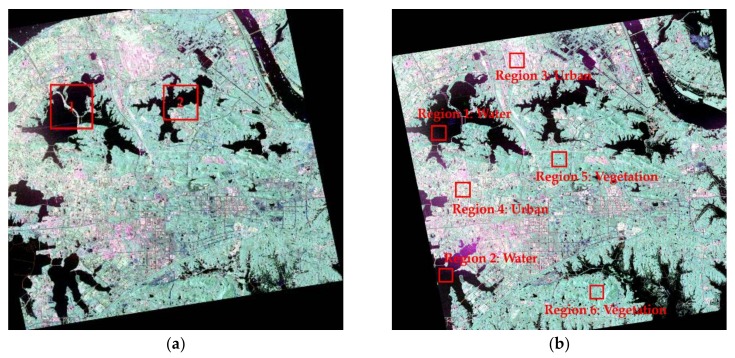
Radarsat-2 polarimetric synthetic aperture radar (PolSAR) images acquired on (**a**) 25 June 2015, and (**b**) 6 July 2016.

The Pauli-RGB images (|*S_hh _*– *S_vv_*| for red (R), |*S_hv_*| for green (G), and |*S_hh _*+ *S_vv_*| for blue (B)) of Wuhan in 2015 and 2016 are shown in Figure 3. In order to verify the efficiency of the proposed method, the regions labeled by the two red boxes (region 1 is East Lake and region 2 is Yanxi Lake) in Figure 3a were selected to provide a detailed assessment. These regions cover urban areas, water bodies, forest, and other urban facilities.

**Figure 4:**
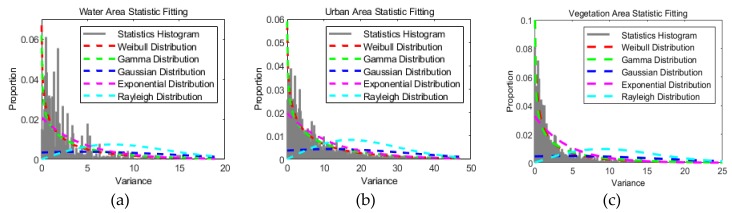
The statistical histograms and distribution function fitting in (**a**) a water area, (**b**) an urban area, and (**c**) a vegetated area.

To establish which distribution model is most compatible with the data, three typical surface features were chosen to analyze the statistical distribution of the DI map: water, a vegetated area, and an urban area. The exact regions are shown in Figure 3b. The statistical histograms of the chosen regions in the DI map are shown in Figure 4. In addition, distribution models based on Rayleigh, exponential, Gaussian, Weibull, and gamma distributions are denoted by the different color curves in Figure 4, and these curves were fitted by the MLE method with pixels in these regions. In Figure 4, it is apparent that the Weibull distribution (red curve) and gamma distribution (green curve) are more suitable than the other function distributions in the water region (Figure 4a), urban region (Figure 4b), and vegetated region (Figure 4c). 

### 4.1. Analysis of the Urban Facilities Changes for East Lake from 2015 to 2016

**Figure 5:**
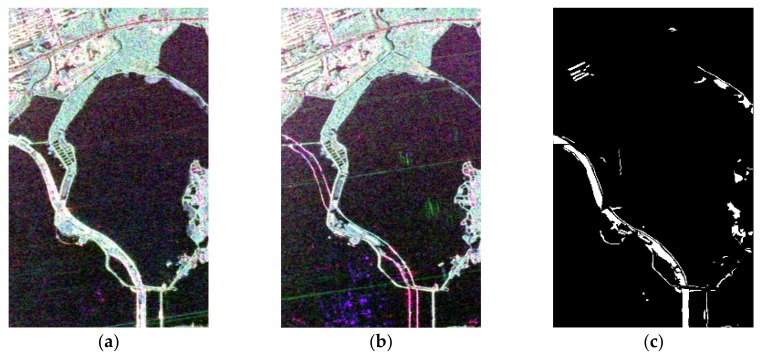
Radarsat-2 PolSAR images of East Lake (region 1) acquired on (**a**) 25 June 2015, and (**b**) 6 July 2016. (**c**) The ground reference map (white denotes change and black denotes non-change).

The first research region was East Lake, for which the image size was 800 × 500 pixels. The land cover of this region is mainly greenway, urban areas, water bodies, and grassland. Due to the greenway re-construction that took place in this period, we focus on a detailed assessment of this area. The Pauli-RGB images at the different dates and the reference data are shown in Figure 5. Furthermore, the reference map was produced by field surveys conducted and visual interpretation from Google Earth.

**Figure 6:**
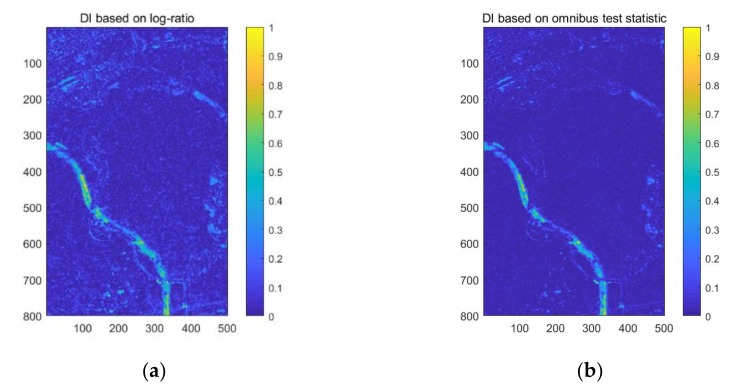
Difference image (DI) maps based on (**a**) the log-ratio method, and (**b**) the omnibus test statistic using PolSAR data from the different dates of 25 June 2015 and 6 July 2016.

The two DI maps generated by the log-ratio method using single-channel SAR information and the omnibus test statistic method using fully polarimetric information are shown in Figure 6. Both of these methods have high values in changed regions. However, the DI map based on the log-ratio method also has a high response in the unchanged areas, which increases the difficulty of distinguishing changed and unchanged regions. Unlike the log-ratio method using single-channel SAR data, the omnibus test statistic method employs more polarimetric information to obtain the DI map, which results in a low response in the unchanged areas. Therefore, the contrast between unchanged and changed regions when using the proposed method is more obvious than for the log-ratio method. It is obvious from Figure 6a, b that the proposed method can obtain a DI map that is more stable and precise. 

**Figure 7:**
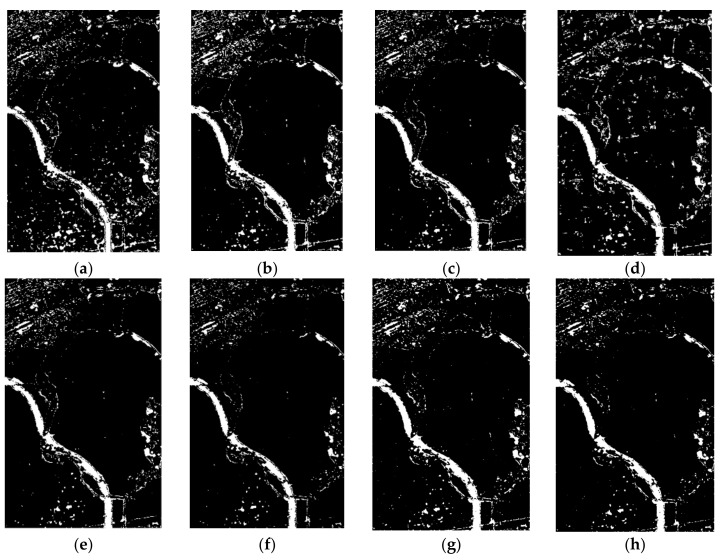
Change detection results: (**a**) log-ratio and Kittler and Illingworth (K&I) algorithm; omnibus test statistic with (**b**) a 5% significance level, and (**c**) a 1% significance level; (**d**) omnibus test statistic and constant false alarm rate (CFAR) algorithm; omnibus test statistic and K&I based on (**e**) a Gaussian distribution, (**f**) a generalized Gaussian distribution, (**g**) a Weibull distribution, and (**h**) a gamma distribution.

The results of the comparison experiment based on the different methods are shown in Figure 7. Because the contrast of the DI map obtained using the single-channel SAR data is not obvious, the result obtained using the log-ratio and K&I method has many wrong detections in the unchanged regions (e.g., water body). Comparing Figure 7a, e, the result based on the omnibus test statistic and K&I method shows a better performance than the result of the log-ratio and K&I method. This proves that using more polarization information from different channels of PolSAR can obtain a DI map that is more accurate and has greater contrast. In order to show the effects of the different threshold selection methods, the methods based on significance levels of 5% and 1%, the constant false alarm rate (CFAR) algorithm, and the traditional K&I algorithm were used in the contrast experiment. The results of the change detection (Figure 7b, c, d, e) demonstrate that using the traditional K&I method results in fewer false alarms than the other methods. Although the traditional K&I method shows a better performance, the Gaussian assumption only fits a symmetric distribution, and the DI map obtained using the fully polarimetric information and omnibus test statistic method does not fit the symmetric distribution assumption. The change detection maps based on the different distribution fitting methods for K&I are shown in Figure 7e–h. Comparing Figure 7e, f, due to one more parameter being used, the K&I method based on a generalized Gaussian distribution obtains fewer false detections in the East Lake area, and it obtains more detail information in the greenway area than the K&I method based on a Gaussian distribution. This proves that adding one more parameter in the Gaussian model can improve the accuracy and efficiency of the change detection results. However, the generalized Gaussian assumption, as an extension of the Gaussian distribution, is still designed for a symmetric distribution. Due to this drawback, both the Gaussian and the generalized Gaussian distributions are not a good fit for the DIs from PolSAR images. This results in the K&I method being unable to accurately extract the optimal threshold. Comparing Figure 7f a, g, the result for the gamma distribution shown in Figure 7h shows the best performance, with fewer false alarms and wrong detections than the other function distributions. Moreover, the K&I method based on a Weibull distribution also performs better than the Gaussian and generalized Gaussian distributions. This proves that, to generate a change detection result with more accuracy and efficiency, choosing a suitable function distribution is more important than adding more parameters. The quantitative evaluation of these different methods is given in Table 2, where the proposed method shows the best performance in all four indicators (FA (%), TE (%), OA (%), Kappa). This confirms that our method is superior to the other methods. 

**Figure 8:**
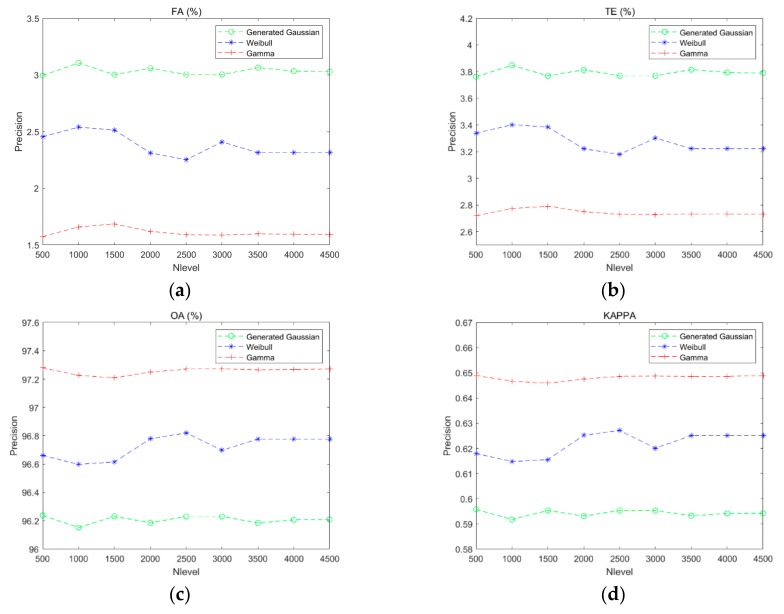
Curves of performance evaluation based on different gray levels: (**a**) false alarm rate; (**b**) total errors; (**c**) overall accuracy; (**d**) Kappa coefficient.

Figure 8 shows the effect of the gray level on the K&I algorithm based on different function distributions. Because the generalized Gaussian distribution is similar to the Gaussian distribution and uses only one more parameter, the generalized Gaussian distribution, the Weibull distribution, and the gamma distribution were used to verify the performance of the proposed method. The different function distributions are shown in Figure 8 by the green curve (generalized Gaussian distribution), blue curve (Weibull distribution), and red curve (gamma distribution). In these figures, it can be seen that the generalized Gaussian distribution obtains the best performance when the gray level is equal to 2500, but the precision is lower than our method based on Weibull or gamma distribution. Furthermore, Weibull distribution is easily affected by the different gray levels, and it obtains the best performance when the gray level is equal to 2500. All the results indicate that the proposed method with gamma distribution in different gray levels performs better than the K&I method with other function distributions. The proposed method is also more stable. 

### 4.2. Analysis of Inundation and Urban Facilities Changes for Yanxi Lake from 2015 to 2016

**Figure 9:**
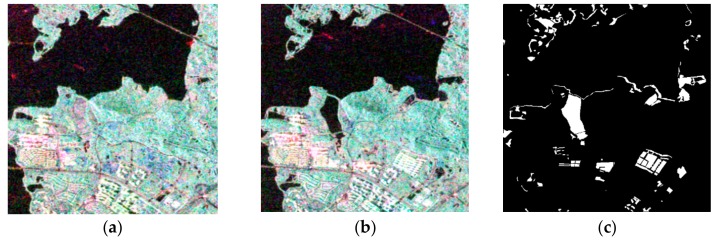
Radarsat-2 PolSAR images of Yanxi Lake (region 2), as indicated in Figure 3, acquired on (**a**) 25 June 2015, and (**b**) 6 July 2016. (**c**) Ground reference map.

The second research region was Yanxi Lake, for which the image size was 600 × 600 pixels. The land cover of this region is mainly urban areas, water bodies, and grassland. Due to the impact of continuous heavy rainfall and construction, the main changes in this area were in the water areas and urban areas. The Pauli-RGB images for the different dates and the ground reference data are shown in Figure 9.

**Figure 10:**
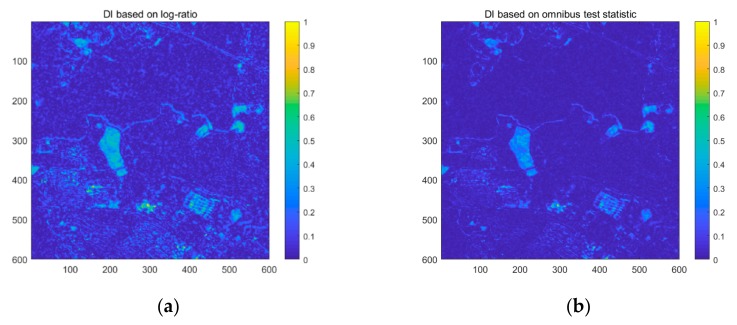
DI maps of region 2 based on (**a**) the log-ratio method, and (**b**) the omnibus test statistic for the different dates of 25 June 2015 and 6 July 2016.

The two DI maps generated by the log-ratio method using single-channel SAR information and the omnibus test statistic method using fully polarimetric information are shown in Figure 10. Both of these methods have high values in changed regions. However, the DI map based on the log-ratio method also shows a high response in the unchanged areas, which increases the difficulty of distinguishing changed and unchanged regions. Unlike the log-ratio method using single-channel SAR data, the omnibus test statistic method uses more polarimetric information to obtain the DI map, which results in a low response in the unchanged areas. Therefore, the contrast between the unchanged and changed regions is more obvious than when using the log-ratio method. Moreover, the visual performance shown in Figure 10a,b proves that the proposed method can obtain a DI map that is more stable and precise. 

**Figure 11:**
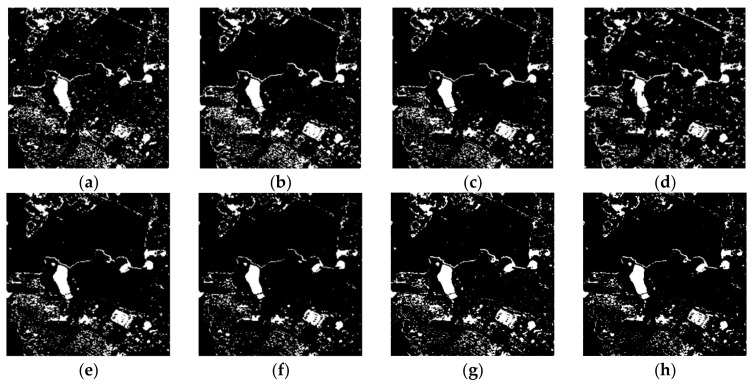
Change detection results: (**a**) log-ratio and K&I; omnibus test statistic with (**b**) a 5% significance level, and (**c**) a 1% significance level; (**d**) omnibus test statistic and CFAR; omnibus test statistic and K&I based on (**e**) a Gaussian distribution, (**f**) a generalized Gaussian distribution, (**g**) a Weibull distribution, and (**h**) a gamma distribution.

The results of the comparison experiment based on the different methods are shown in Figure 11. Comparing Figure 11a, e, the results based on the omnibus test statistic and K&I method show a better performance than the results of the log-ratio and K&I method. This again proves that using more polarization information from different PolSAR channels can obtain a DI map that is more accurate and has greater contrast. The results of the change detection (Figure 11b–h) show that using the K&I method with different distribution results in fewer false alarms than for the other methods. Moreover, the results based on the proposed method obtains fewer false detections in the Yanxi Lake area, and it obtains more detailed information in the urban area. This again proves that, to produce a change detection result with more accuracy and efficiency, choosing a suitable function distribution is more important than adding more parameters. The quantitative evaluation for the different methods is provided in Table 3, where the proposed method based on gamma distribution shows the best performance in all four indicators (FA (%), TE (%), OA (%), Kappa). Moreover, Weibull distribution also shows a better performance than the Gaussian and generalized Gaussian distributions. This confirms that the proposed method is effective and shows a significant improvement in detecting the changes caused by urbanization and inundation over the other methods. 

**Figure 12:**
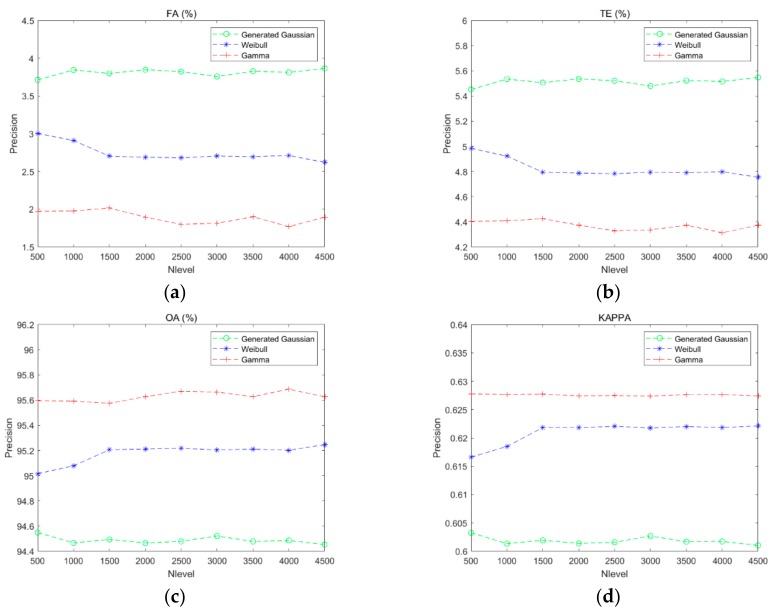
Curves of the performance evaluation based on different gray levels: (**a**) false alarm rate; (**b**) total errors; (**c**) overall accuracy; (**d**) Kappa coefficient.

Figure 12 shows the effect of the gray level on the K&I algorithm based on the different function distributions. In the figures, it can be seen that the generalized Gaussian distribution obtains the best performance when the gray level is equal to 3000, but the precision is lower than the other function distributions. The Weibull distribution is stable when the gray levels are above 1500. These results indicate that the proposed method with gamma distribution performs better than the K&I method with other function distributions for the case of different gray levels. Again, the proposed method is also more stable than the other methods.

## 5. Discussion

**Figure 13:**
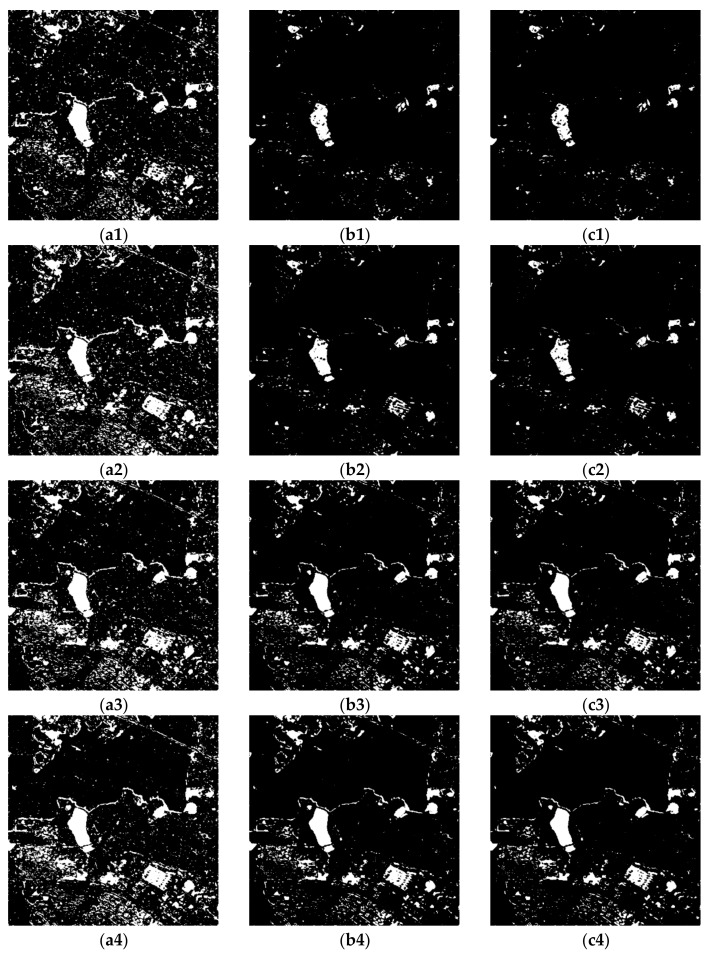
Change detection results using the HH polarization based on omnibus test statistic and K&I based on (**a1**) a generalized Gaussian distribution, (**b1**) a Weibull distribution, and (**c1**) a gamma distribution; the VV polarization based on omnibus test statistic and K&I based on (**a2**) a generalized Gaussian distribution, (**b2**) a Weibull distribution, and (**c2**) a gamma distribution; the (HH, HV) polarization based on omnibus test statistic and K&I based on (**a3**) a generalized Gaussian distribution, (**b3**) a Weibull distribution, and (**c3**) a gamma distribution; the (VV, VH) polarization based on omnibus test statistic and K&I based on (**a4**) a generalized Gaussian distribution, (**b4**) a Weibull distribution, and (**c4**) a gamma distribution.

**Figure 14:**
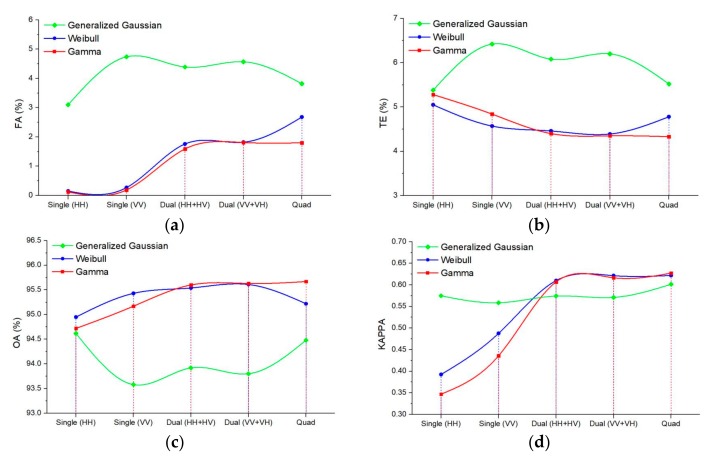
Quantitative evaluation of results from different polarizations: (**a**) false alarm rate; (**b**) total errors; (**c**) overall accuracy; (**d**) Kappa coefficient.

In this section, the potentials and limitations of the proposed method are discussed. The proposed method using multi-temporal PolSAR data has better performance than other methods. In order to verify the usability of our approach, we made further experiments based on multi-temporal single and dual polarization data in Yanxi lake. Single and dual polarization information extracted from the complex target vector in Equation (1) are applied in the proposed method. The change detection results in Yanxi Lake are shown in Figure 13. The results illustrate that the proposed method can also be applied in single or dual polarization data. Moreover, the results of dual polarization data based on Weibull (Figure 13b3, b4) or gamma distribution (Figure 13c3,c4) have good performance. However, compared with the results of dual polarization, the results of single-channel SAR data have more omission detection in Figure 13b1(2), c1(2). This proves that using more polarization channel information can obtain more accurate change detection results. Comparing Figure 13b1(2), c1(2), the results with single polarization data based on generalized Gaussian distribution have a better performance in Figure 13a1(2). This proves that the symmetric distribution assumption is the most compatible with DI map from the single polarization. Furthermore, quantitative evaluation of results from different polarizations is provided in Figure 14 by the green curve (generalized Gaussian distribution), blue curve (Weibull distribution), and red curve (gamma distribution). The proposed method based on gamma distribution using quad polarization data shows the best performance in TE, OA, and Kappa than others. Moreover, the accuracy (red curve) improves as the polarization increases in Figure 14b–d. This confirms that using more channel information produces more stable and precise results. In addition, the proposed method based on Weibull distribution (blue curve) with dual polarization data has better performance (TE and OA) than single and quad polarization. Although the Kappa is lower than that from quad polarization, the difference is small. These demonstrate that the proposed method based on Weibull distribution is suitable in dual polarization. It proves that our approach has great potential to be applied in Sentinel-1 data with dual polarization datasets. 

Although the proposed method has a good performance in change detection and can be applied in both single and dual polarization data, it still has some limitations. Firstly, the proposed method can detect the water changes in two dimensions, but it cannot detect the water-level changes. To solve this problem, external data are applied to improve the change detection accuracy. Secondly, the proposed method can obtain the unchanged and changed information, but it cannot obtain the types of land-cover changes. To solve this problem, classification techniques can be combined with our method to get the types of land-cover changes. Thirdly, multi-temporal time-series observations are ideal for detecting changes accurately. Because of limited data, our research only uses the bi-temporal PolSAR data to detect the changes in urban facility and inundation. The application of the proposed method based on time-series observations from different sensors (e.g., Sentinel-1) will be explored in further work.

## 6. Conclusions

In this paper, we showed that the combination of an omnibus test statistic using a covariance matrix and the K&I method based on Weibull or gamma distribution can improve the performance of change detection for multi-temporal PolSAR images. The proposed method for generating DI maps is appropriate for PolSAR images, and it can obtain an accurate intermediate result. Due to the solid mathematical fundamentals of Bayesian theory, the traditional K&I method shows better performance than the other methods. This illustrates that the threshold selection method based on K&I is stable and effective in change detection. After analyzing three typical land covers, i.e., water, vegetation, and urban areas, in the difference image, we find that the variable in the difference image more likely follows the Weibull or gamma distribution. Thus, we proposed the K&I change detection algorithm based on Weibull or gamma distribution. Moreover, the proposed method can be applied to single/dual/quad polarization data, while the different polarization data with different distributions can generate stable and precise change detection results. Based on the proposed method, the changes caused by urban development and inundation between 2015 and 2016 in Wuhan, China, were detected accurately. Over the East Lake and Yanxi Lake regions where the reference map was available, our proposed method obtained the best performance with FA of 1.59% and 1.80%, TE of 2.73% and 4.33%, OA of 97.27% and 95.67%, and Kappa of 0.6486 and 0.6275, respectively. These confirmed that the proposed method is superior to the other methods, and it is effective in detecting changes caused by urbanization and inundation.

## Figures and Tables

**Table 1:** Detailed parameters of the test datasets.

Date of Observation	Beam Mode	Pixel Spacing (m) Azimuth × Range	Incidence Angle (°)	Swath Width (km)
25 June 2015	FQ21	5.12 × 4.73	40.16–41.58	25 × 25
6 July 2016	FQ27	4.86 × 4.73	45.23–46.69	25 × 25

**Table 2:** Performance evaluation of the change detection for East Lake. FA—false alarm; TE—total error; OA—overall accuracy.

Method	FA (%)	TE (%)	OA (%)	Kappa
Log-ratio and traditional K&I	6.55	7.03	92.96	0.4413
Omnibus test statistic with a 5% significance level	6.17	6.42	93.58	0.4857
Omnibus test statistic with a 1% significance level	4.59	5.04	94.96	0.5398
Omnibus test statistic and CFAR	6.53	6.84	93.16	0.4630
Omnibus test statistic and traditional K&I	3.96	4.51	95.49	0.5629
Omnibus test statistic and K&I based on a generalized Gaussian distribution	3.00	3.77	96.23	0.5953
Omnibus test statistic and K&I based on a Weibull distribution	2.25	3.18	96.82	0.6271
Omnibus test statistic and K&I based on a gamma distribution	1.59	2.73	97.27	0.6486

**Table 3:** Performance evaluation of the change detection for Yanxi Lake.

Method	FA (%)	TE (%)	OA (%)	Kappa
Log-ratio and traditional K&I	6.69	7.97	92.03	0.5135
Omnibus test statistic with a 5% significance level	7.22	7.99	92.01	0.5358
Omnibus test statistic with a 1% significance level	5.37	6.57	93.43	0.5749
Omnibus test statistic and CFAR	6.70	7.92	92.08	0.5179
Omnibus test statistic and traditional K&I	4.86	6.19	93.81	0.5862
Omnibus test statistic and K&I based on a generalized Gaussian distribution	3.82	5.52	94.48	0.6016
Omnibus test statistic and K&I based on a Weibull distribution	2.68	4.78	95.22	0.6220
Omnibus test statistic and K&I based on a gamma distribution	1.80	4.33	95.67	0.6275
